# Bacteria Delay the Jamming of Particles at Microchannel Bottlenecks

**DOI:** 10.1038/srep31471

**Published:** 2016-08-11

**Authors:** Zenamarkos Bantie Sendekie, Arthur Gaveau, Rob G. H. Lammertink, Patrice Bacchin

**Affiliations:** 1 Laboratoire de Génie Chimique, Université de Toulouse, CNRS, INPT, UPS, Toulouse, France; 2 Soft Matter, Fluidics and Interfaces, MESA+ Institute for Nanotechnology, Faculty of Science and Technology, University of Twente, Enschede, The Netherlands

## Abstract

Clogging of channels by complex systems such as mixtures of colloidal and biological particles is commonly encountered in different applications. In this work, we analyze and compare the clogging mechanisms and dynamics by pure and mixture suspensions of polystyrene latex particles and *Escherichia coli* by coupling fluorescent microscopic observation and dynamic permeability measurements in microfluidic filters. Pure particles filtration leads to arches and deposit formation in the upstream side of the microfilter while pure bacteria form streamers in the downstream zone. When mixing particle and bacteria, an unexpected phenomenon occurs: the clogging dynamics is significantly delayed. This phenomenon is related to apparent “slippery” interactions between the particles and the bacteria. These interactions limit the arches formation at the channels entrances and favour the formation of dendritic structures on the pillars between the channels. When these dendrites are eroded by the flow, fragments of the deposit are dragged towards the channels entrances. However, these bacteria/particles clusters being lubricated by the slippery interactions are deformed and stretched by the shear thus facilitating their passage through the microchannels.

When flowing particles through a constriction, different mechanisms (arches formation^1^, finger-like structure^2^, crystallization^3^, jamming, deposition^1^,^3^) can cause a reduction in permeability. Such phenomena have important consequences in medical, biomedical and in numerous industrial applications (filtration, ink jet printing, etc.).

A general way to depict such phenomena is still lacking. One of the main reason is the crucial role played by surface interactions in these phenomena (even for microparticles that are usually considered as non-colloidal). These multibody particle-particle and particle-wall interactions are adding new important sources of complexity to the problem^4^ that is rather difficult to anticipate.

Collective effects induced by DLVO interaction have been simulated^5^,^6^. The inter-particle repulsion can lead to the formation of a network of interacting particles under flow that can lead to intermittent flow at the bottleneck entrance. Experimentally, it has been shown that the scenarios of microchannel clogging are very different when changing the interparticle interaction profile^7^. These scenarios can be linked to the one observed with other kind of interacting objects (pedestrians, self-propelled particles, granular matter, etc.)^4^. The nature of the multi-body interactions is the key for the description of these problems. It is possible from anthropomorphism consideration^8^ to link arches formation at a bottleneck to a panic scenario due to repulsion and a cooperative transport delaying clogging to a herding scenario due to short-range secondary attraction^7^.

The behaviour of colloidal particles is better understood compared to biological particles like bacteria. When flowing in confined systems, bacteria form filamentous structures known as streamers^9^,^10^ that result in mature biofilm formation and to a rapid clogging of devices^11^. It has been shown that the interaction between bacteria and surfaces are principally dependent to the presence of exopolysaccharides (EPS) in the system.

The clogging behaviour of bacteria and particles is thus completely different: bacterial streamers are filamentous structures that are developed in the downstream zone of a constriction whereas particle clogs are formed in the upstream zone of the constriction. However, there are still open questions about the phenomena and the dynamics when filtering bacteria and particles mixtures: for instance, what is the interplay between biological particles and colloidal particles at a constriction?

In this work, the aim is to provide intimations to answer some of these questions by investigating the clogging of 5 μm width microfluidic channels (Fig. 1) with a dynamic microscopic view of the clogging patterns coupled with dynamic measurement of the permeability variation. Experiments (summarized in Table 1) have been realized with monodisperse polystyrene latex particles (2.3 μm) and fluorescent *Escherichia coli* (1 to 2.5 μm rod shape cells) pure and mixed suspensions. Two concentrations of *E. coli* are considered to analyse the bacterial concentration effect: low concentration (denoted as *E. coli LC*) and high concentration (denoted as *E. coli HC*) as shown in Table 1.

## Results

**Figure 1:**
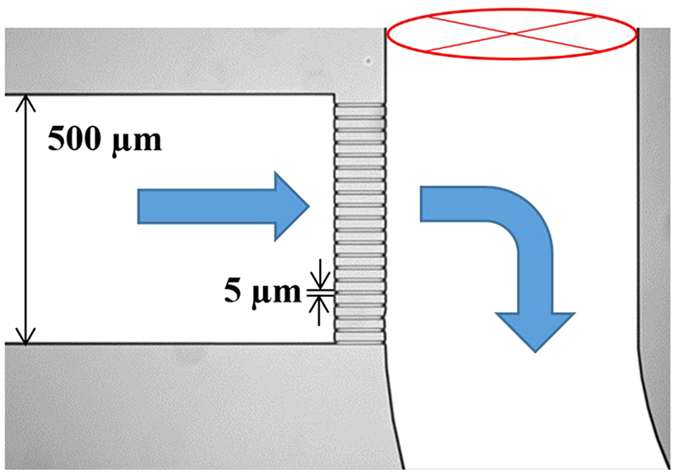
Image of the microfluidic device. The arrows are indicating the flow direction through the array of microchannels (5 μm width) where the filtration and the associated jamming take place.

Filtration experiments are performed using a microfluidic device (Fig. 1) at constant flow rate of 20 μL/min (i.e flow velocity of 0.170 m/s corresponding to a Reynolds number of 1.36). The pressure, flow rate and permeate volume are measured with time which are coupled with images from microscopic observations of the filtration experiments enabling both quantitative and qualitative analyses of the results. Additional information is given in the material and methods section.

### Bacterial streamers versus particles clogs

**Figure 2:**
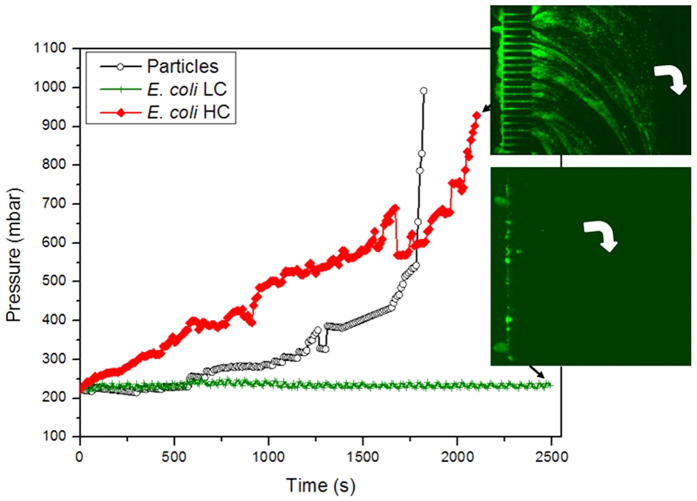
Comparison of the clogging dynamics by polystyrene particles and *E. coli* at different concentrations. The filtration experiments are performed at constant flow rate of 20 μL/min. The inset images are taken at the end of the experiments for the low and high concentration *E. coli* suspensions.

First, the clogging dynamics of different *E. coli* concentration suspensions have been investigated and compared with particles. Two concentrations of *E. coli* are considered (5*10^6^ UFC/mL and 10^8^ UFC/mL). As shown in Fig. 2, no clogging of the filter (or increase in pressure or flow resistance) is observed during filtration of low concentration *E. coli* suspension (5*10^6^ UFC/mL) in a pseudo-cross flow mode in the time frame of the experiments (about two and half hours). After filtration of the bacterial suspensions at high concentration (20 times higher), streamers start to form within a few minutes in the downstream zone and increase rapidly with time in their density and length. From 3D confocal characterization^10^, it appears that the streamers are mainly very long separate jets in morphology without attaching to each other until the end of the experiments. The streamers are oriented in the flow direction due to the change in flow in the downstream side of the filter (pseudo-cross flow in permeate side) and oscillate rapidly in the flow (see inserted images in Fig. 2). The pressure increases rapidly from the initial 200 mbar: it reaches about 950 mbar in about 40 minutes of filtration time. This pressure increase is the consequence of the streamer formation (as can be seen from video 1 in Supplementary Information). This formation of the streamers has been discussed in previous work^9^,^10^ and has been identified as an important cause in flow disruption^11^. There are also some sudden drops in pressure which are due to the detachment of dense streamers by the background fluid flow; these are frequently observed during experiments. The rapid increase in pressure during filtration of high concentration *E. coli* suspensions is due to the higher capture of bacteria in the microchannels and in the streamers formed (the fishing net effect created by the streamers is more pronounced). Previously, A. Marty *et al*.^10^ found that changes in flow direction in tortuous porous media prompted streamer formation. They used the same bacterial suspensions (i.e., *E. coli*) and channel geometry (straight channels) and the same order of magnitude of the flow conditions to our case. They reported that the pseudo-cross flow configuration led to a rapid and greater formation of streamers compared to dead-end flow modes.

The clogging of microchannels by bacteria is completely different to clogging by particles: particles clog the microchannel entrances (the upstream zone of the channels) leading to dense deposit formation. It has been shown that particle-particle and particle-wall DLVO interactions are controlling the dynamics of the formation and the breakage of these clogs^7^. Detailed analysis of the clogging dynamics of colloidal particles (polystyrene latex particles) showed^7^ that rapid clogging of microchannels occurs at coupled filtration conditions of high ionic strength (100 mM KCl) and intermediate flow rate (30 μL/min) and the clogging is significantly delayed at moderate ionic strength (10 mM), the low repulsion barrier and the secondary minima playing important roles, respectively. In this report, the clogging dynamics of the same particles is analysed using NaCl to vary the ionic strength (154 mM), i.e. at the same ionic strength with the one needed for isotonic bacterial suspension. Consistent with previous results^7^, rapid clogging of the microchannels is observed during filtration of polystyrene suspensions at high ionic strength (154 mM) (Fig. 2): the pressure reaches 1 bar in 33 minutes.

In contrary, the bacterial suspension at identical volume fraction with particles (i.e., 5*10^6^ UFC/mL corresponding to a volume fraction of 10^−5^) does not demonstrate any clogging even at longer filtration time, 42 minutes. Based on these strong differences in clogging mechanisms and dynamics, the next section investigates the filtration of mixed particles and bacteria suspensions.

### Bacteria & Particles clogging

**Figure 3:**
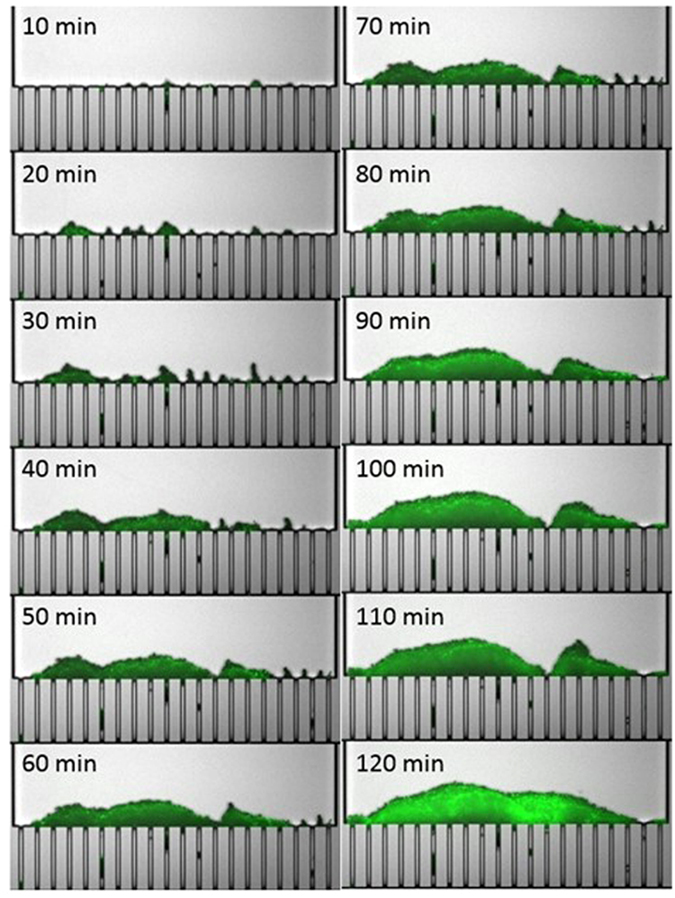
Temporal evolution of the clogging phenomena during filtration of mixed polystyrene particles and *E. coli* (at 5*10^6^ UFC/mL concentration) suspension. The filtration experiment is performed at constant flow rate of 20 μL/min.

Figure 3 shows microscopic observation of the evolution of the clogging during filtration of mixed *E. coli* and polystyrene suspension. It shows merged images of green fluorescence (using SYTO 9 green fluorescent to observe *E. coli*) and the yellow channel (with no fluorescence) in order to observe both the particles and the bacteria at the same time. In comparison with the clogging phenomena with the pure suspensions, different clogging mechanism has been observed during filtration of mixed suspensions. Frequent formations and collapses of dendritic structures on the pillars of the microchannels are observed for the mixed suspensions (see video 2 in Supplementary Information). Furthermore, no streamer formation in the downstream side of the microchannels is observed but bacterial cells are captured in the upstream side of the microchannels in connexion with the particles. Thus, the presence of *E. coli* in the suspension results in a different clogging mechanism.

**Figure 4:**
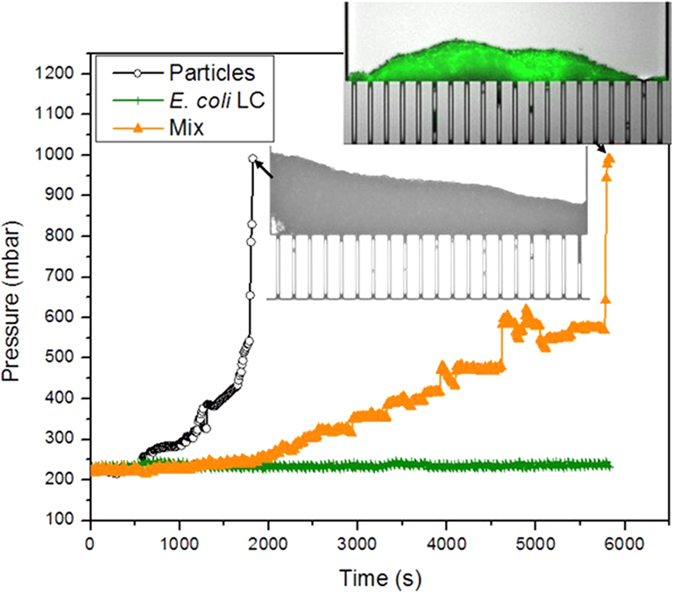
Comparison of the clogging dynamics of suspensions of polystyrene particles, *E. coli,* and mixture of the two. All the experiments are performed at constant flow rate of 20 μL/min. The volume fractions of the particles and bacteria are the same (10^−5^).

Figure 4 shows comparison of the clogging dynamics of *E. coli*-only and polystyrene-only suspensions with the mixture of the two at the same volume fraction and ionic strength (Table 1). It can be clearly seen from Fig. 4 that the clogging dynamics of polystyrene particles is significantly modified by the presence of *E. coli* in the suspension: a substantial delay in clogging occurs. The microseparator is completely clogged (the pressure reaches the maximum value ≈1 bar) after about 6000 seconds during filtration of the mixed suspension while the corresponding time for particles-only suspension is about 1800 s. There are also small fluctuations in pressure during filtration of the heterogenic mixture. As indicated above, this is because of the frequent formation and collapse of dendrites (that form on the pillars between two channel openings) and their subsequent removal through the microchannels. Thus, the presence of *E. coli* in polystyrene suspension results in a delay in initiation of the clogging and in the formation of more fragile dendrites. The relative kinetics of particle and bacteria accumulation obtained from image analysis is presented in Figure S2 in Supplementary Information. From this analysis, it can be noticed that the particle deposition is prevalent to the bacteria deposition. Furthermore, the first deposition of particles and bacteria (before 2000 s) having dendritic shape is not inducing a significant hydraulic resistance. It can also be noted (Figure S3 in Supplementary Information) that the green signal (due to the bacteria presence) is very well correlated to the pressure increase due to the clogging (Fig. 4). This correlation could be due to the fact that the capture of the bacteria in the particle deposit is directly linked to the percolation of the water through the deposit.

### Bacteria delay particle clogging

**Figure 5:**
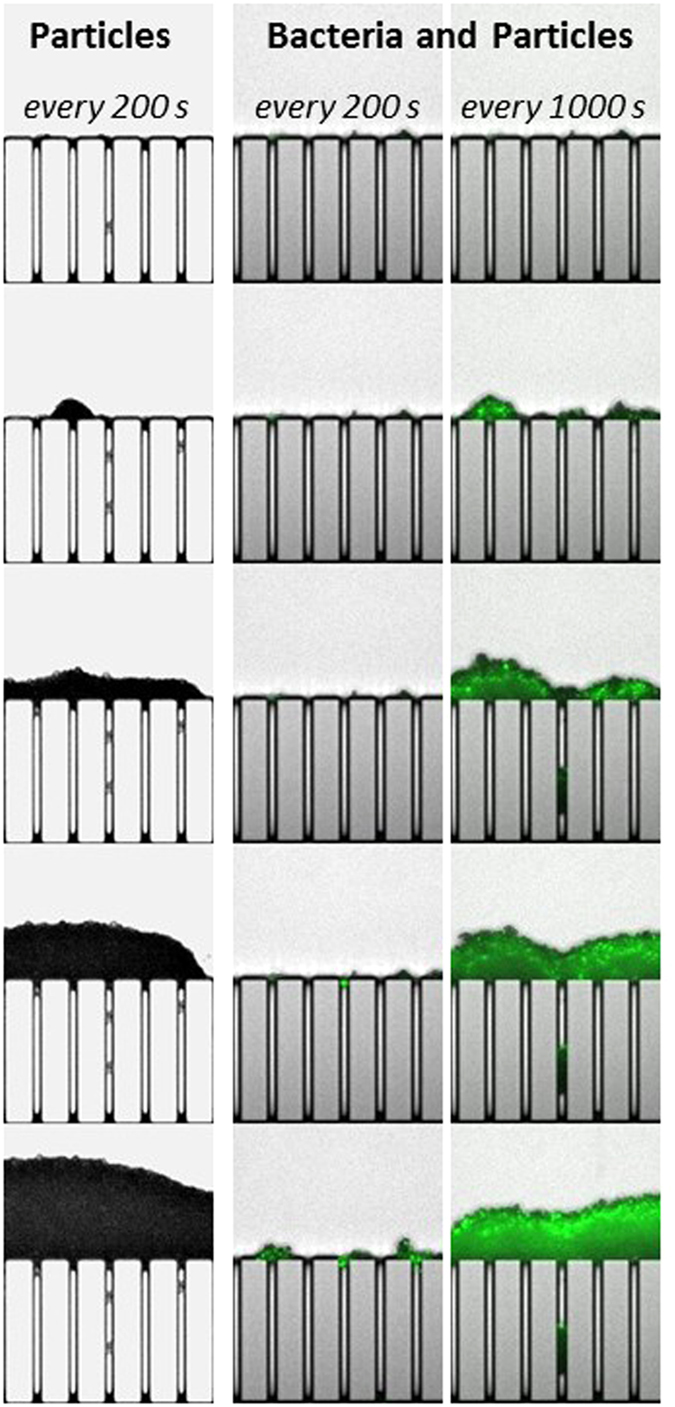
Illustration of the clogging kinetics differences between the particles (left column) and the particle/bacteria mixture (two right columns) filtrations. When comparing the first two columns (with a same time scale), it is possible to see that the clogging is leading to a dense deposit (for particles filtration) while almost no accumulation is observed for the mixture. It can be seen that the clogging kinetics is almost the same when comparing the first column for particles and the third column for the mixture (the time scale is augmented by 5).

When bacteria (with a volume fraction of 10^−5^) is added to a dispersion of particles (with a volume fraction of 10^−5^), the clogging is appearing later compared to a particles-only dispersion (with the same volume fraction, 10^−5^). These results are rather counter-intuitive for two reasons: i) the mix is more concentrated (the total volume fraction is 2*10^−5^) but leads to less clogging ii) the bacteria should induce some additional stickiness in particle-particle and particle-wall interactions that should favour the clogging. The kinetics difference is illustrated in the Fig. 5 where it can be seen that the clogging kinetics is around 5 times slower for the mixture.

## Discussion

### Bacteria or EPS reduce the capture efficiency of particles or particles/bacteria aggregates

**Figure 6:**
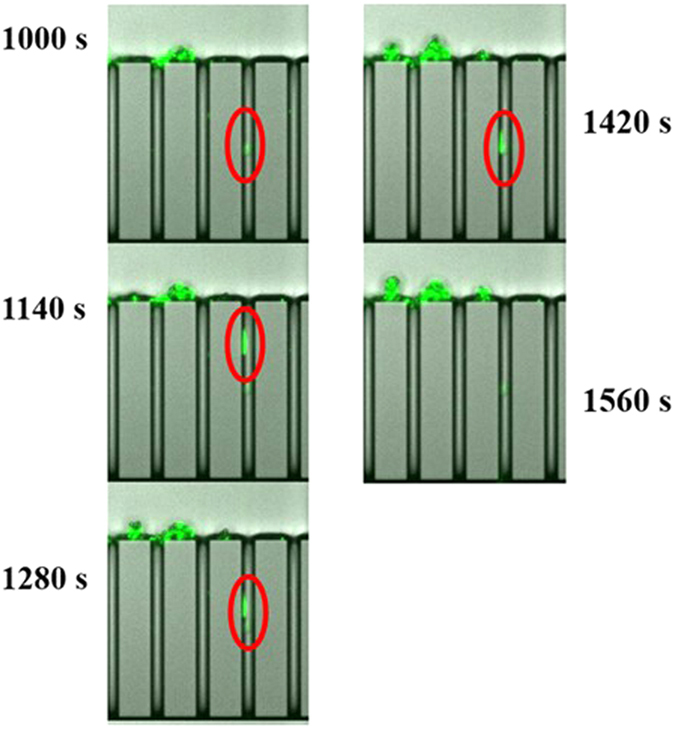
Observation of a bacterial aggregate gliding inside a microchannel (the gliding velocity is estimated at 2*10^-7^ m/s m/s, so well lower than the fluid velocity 0.17 m/s). This bacterial accumulation do not induce particles capture.

It seems that the presence of bacteria or EPS in a particles suspension is limiting the capture efficiency of particles: i) particle capture is delayed, ii) particle aggregates detached from the dendrites are swept out through the channel. Fig. 6 illustrates the gliding of a particle/ /bacteria/EPS aggregate along the channel wall before being finally swept out from the channel.

The specific surface interaction displayed by bacteria at walls have been discussed in several papers. The local increase in viscosity at the channel walls or the presence of polymer brush due to the presence of EPS could slow down the bacteria/wall collisions and then the bacteria attachment at the wall. Elimelech *et al*.^12^ reported the deposition of bacterial cells (*E. coli*) in the secondary energy minimum at moderate to high ionic strengths (10–300 mM KCl). These secondary minimum interactions could help the bacteria to translate or roll along the microchannel wall due to fluid drag and shear as demonstrated on collectors^13^. A huge delay in clogging has also been observed when filtering particles exhibiting secondary minimum conditions through microchannels^7^. Thus, the retardation in the clogging dynamics during filtration of mixed *E. coli* and polystyrene suspensions could be due to a gliding effect of bacteria or particle/bacteria aggregate via secondary energy minimum interaction with the wall. Such short-range secondary minimum attraction could not only be due to DLVO interactions but also to more complex interactions involving EPS such as depletion attractions. In the case of aggregation mechanisms of particles having secondary minimum interaction, these interactions have been defined as slippery bonds^14^ that are not shear-rigid and permit translational or rotational movement over a surface in contrast to shear-rigid bonds due to frictional force at contact.

**Figure 7:**
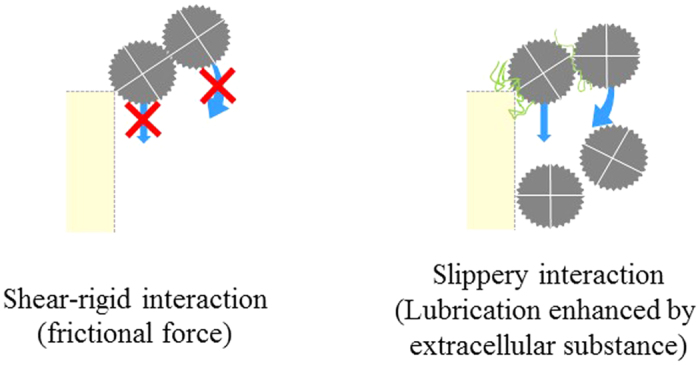
The presence of bacteria and extracellular polymeric substances can induce particle/particle and particle/wall slippery interactions. These slippery interactions can permit translational or rotational movement (the scheme on the right) thus preventing the formation of arches that can appear for shear-rigid interaction (the scheme on the left).

The slippery interactions can reduce the capture of an isolated particle by favouring the gliding of particle on the channel wall and, for numerous particles, could prevent the formation of arches at channels entrances by favouring the rotational movement between particles (Fig. 7). The inter-particle frictional force has been recently shown^15^ as the predominant mechanism to explain the jamming transition: the viscosity divergence when increasing the shear (shear-thickening). The clog formation and the shear-thickening have the same physical origin: the formation of force chains^16^ between the particles. As the shear rate increases in rheology measurement (or when the time increases during the clogging), more inter-particle contacts form leading to frictional forces that can percolate in the system resulting in force chains. If the local force between the particles are less frictional, the jamming point, where contact forces dominate the macroscopic properties, appears for higher volume fraction or higher shear rates^15^. The essential role of the frictional force on the shear-thickening can explain why the lubrication (may be due to the extracellular polymeric substances) when mixing bacteria and particles could delay the clogging. Additionally, the existence of attractive interaction between particles and bacteria could also induce a collective herding instinct scenario^8^ that could favour the cooperative transport of the particles through the channels. These specific slippery particle/bacteria/EPS/wall interactions could have different physical origins that are not totally unravelled yet.

### The clogging morphology is different when bacteria are added to particles

**Figure 8:**
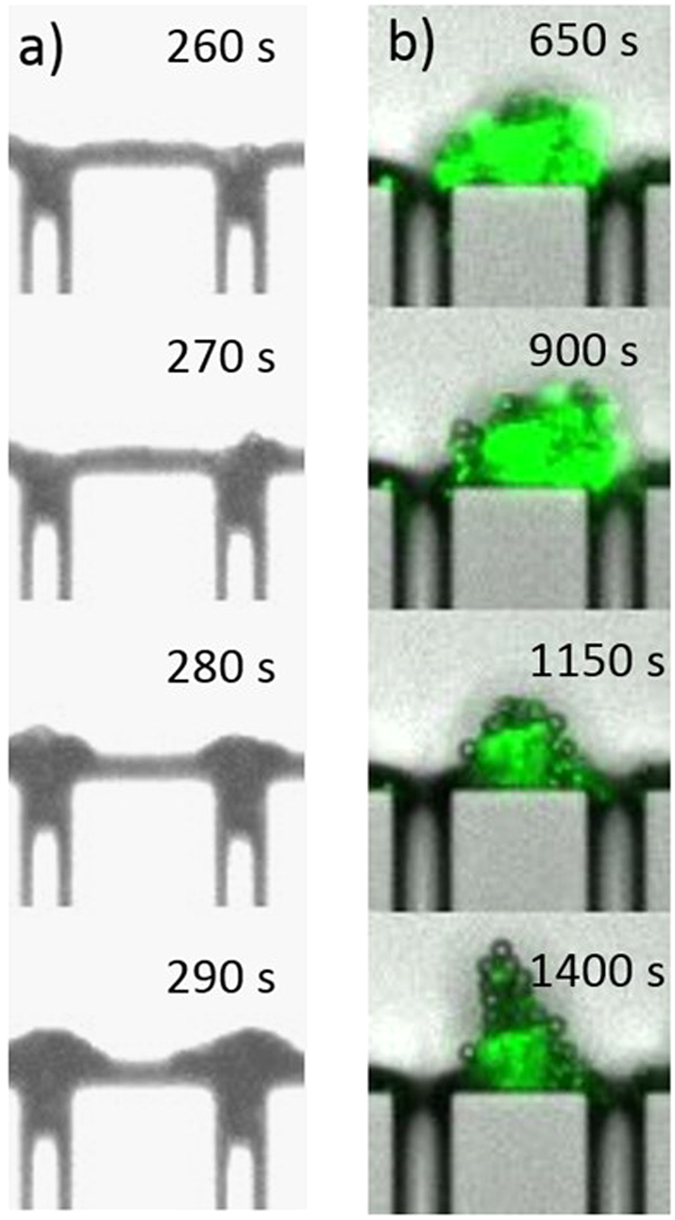
Morphologies of the initial mass accumulation at bottlenecks. (**a**) for particles, arches form at microchannel openings leading to the formation of clogs blocking the entrances. (**b**) for particles/bacteria mixture, dendrites formation on the pillars between the microchannels openings (when broken or eroded, the fragments are swept out through microchannels).

When zooming on a bottleneck at the initial steps of clogging (Fig. 8), the morphology of the mass accumulation is characterized by arches formation leading to the microchannel clogging for particles whereas dendrites are formed on the pillars between the microchannel entrances for the mixture.

With particles, the clogs can be sometimes swept out by the flow (exhibiting some fragility) however their re-formation is very rapid leading to the progressive formation of dense deposit when the clogs are joining (Fig. 8a). With the particle/bacteria mixture, the clogging is initiated with accumulation on the pillars (Fig. 8b). Such a difference between arches and dendrites formation could be the consequence of different positioning of the particles at the channel corners. It has been shown that the capture position of particles at a pore corner (that could initiate arches) could result from a torque balance between drag forces and van der Waals interaction^17^. The presence of specific interactions, like slippery ones, could affect this balance and favour a more central positioning on the pillars^18^ leading to dendrites formation. Furthermore, it has been shown that the formation of arches is resulting from the establishment of force chains between particles^16^. These force chains can be more resistant when particles are interacting by frictional forces at contact (for pure particles) than in presence of slippery interactions (precluding shear-rigidity) between particles (for bacteria/particle mixing). The force chains could have a low probability to form at the pore bottlenecks. It can also be observed in Fig. 7b that the shape of dendrites is changing a lot with time proving that some erosion of the dendrites takes place. The erosion has been already discussed as a possible mechanism to explain finger-like structure^2^.

### Fragility induced by bacteria

**Figure 9:**
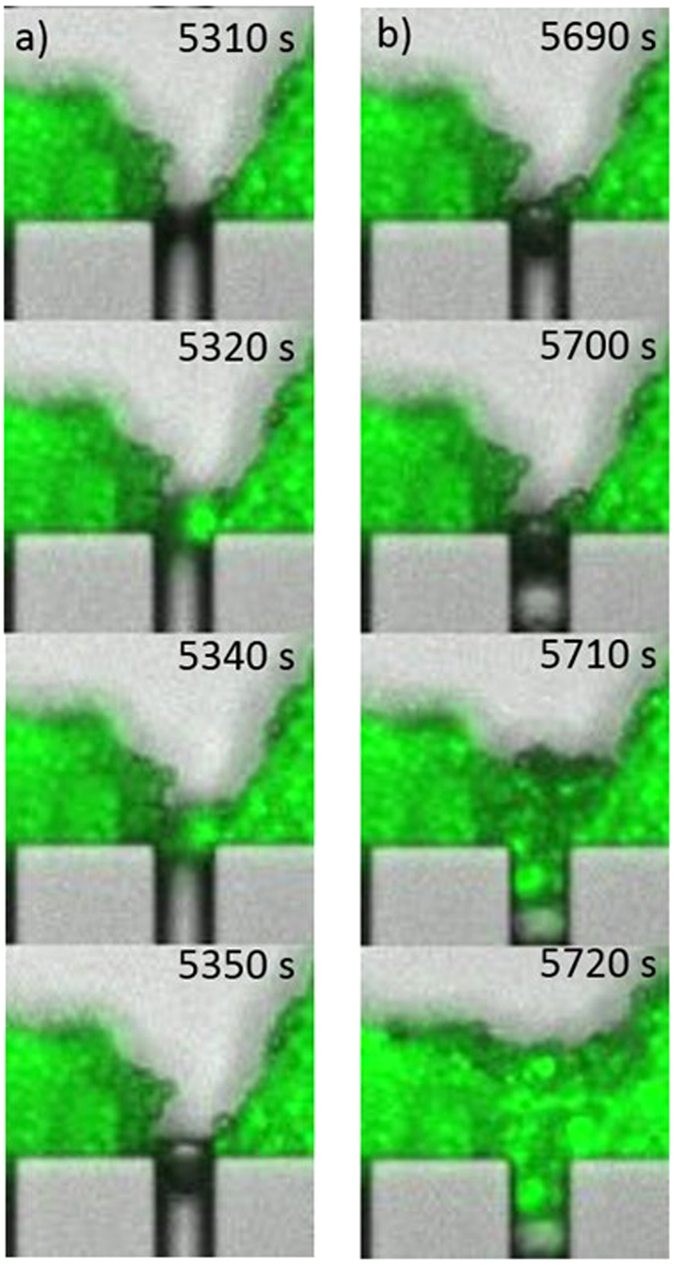
Dynamics of blocking and reopening events. Particle/bacteria aggregate coming from the erosion of dendrites can be swept out with the flow if the fragments are small (**a**) or can lead to the permanent obstruction of the channels if the fragment are larger (**b**).

The bacteria/particle aggregates seem to exhibit fragility. Such fragility is observed for initial dendrites formation (Fig. 8b) whose shape is frequently changing. Another noticeable point is the fact that when particle/bacteria aggregates are detached from dendrites, these fragments are not clogging the channels but quickly drained through. When the deposition is progressing (at longer times, Fig. 9a), erosion of the deposit can lead to larger fragments thus leading to channel obstruction. The obstruction is then inducing a pressure increase (Fig. 4). However, it is possible to observe frequent reopening of the clogged channels (linked to a pressure decrease in Fig. 4).

The particle/bacteria clusters seem then to break or deform by the flow. This fragility could be due to the slippery bonds linking the entities inside the aggregate that could then be seen as a network of lubricated bodies. Because of these slippery bonds, particle/bacteria aggregates can be deformed and stretched thereby facilitating their draining with the flow in the microchannel. Complete clogging of the channels appears only when the fragments are large enough (Fig. 9b) and cohesive enough to resist the flow.

## Conclusions

The clogging dynamics of pure suspensions of polystyrene colloidal particles and *Escherichia coli* bacteria and their heterogenic mixtures have been analysed by both microscopic observations and dynamic measurements of the flow rate and pressure with time. The particles form deposits in the upstream zone of the microseparator in which the clogging is initiated by arches formation at the bottleneck. On the other hand, bacteria form filamentous structures in the downstream zone of the filter. The clogging dynamics of the heterogenic mixtures of the polystyrene colloids and *E. coli* show different mechanisms compared to the dynamics of the pure suspensions. The clogging dynamics is significantly decelerated. This is mainly due to a change in the initial accumulation deposit: arches formation for particles and dendrites formation for particles/bacteria mixture. This difference could be due to the presence of slippery bonds inside the particle/bacteria aggregates. These slippery bonds could change the force balance leading to the arches formation and then promote the formation of dendrites. Furthermore, these slippery bonds could promote the deformability and the stretching of the fragments eroded from the dendrites facilitating their draining through the microchannels. These specific interactions, probably due to bacteria and/or EPS (and previously discussed as short-range secondary attraction), could then explain the clogging delay induced by the presence of bacteria in a particle dispersion.

## Materials and Methods

The microfluidic device used for this work is fabricated by dry reactive-ion etching (DRIE) process from silicon and glass wafers (see Figure S1 in Supplementary Information). The microchannels are constituted of an array of 20 parallel channels having width of 5 μm. The main channel and the microchannels have depth of about 20 μm. The chip is designed for cross flow separation or fractionation of ‘particles’ (biological and colloidal particles) and it is used in a pseudo-cross-flow mode in the permeate side for this experimental work.

*Escherichia coli* strain CIP 54.127 (obtained from the Institute Pasteur collection, Paris, France) is used for this experimental work. The bacterial cells are grown aerobically on a complex medium of tryptone soy agar (Biomérieux). The culture is incubated at 37 °C in stationary condition for 24 hrs. The inoculum is prepared by suspending colonies in sterile physiological solution of 9 gL^−1^ of sodium chloride. The bacterial suspensions are kept in a non-nutritive condition. Concentration at 10^8^ UFC/mL is initially adjusted by optical density measurement at 640 nm and then different concentrations of *E. coli* (ranging from 5*10^6^ to 10^8^ cells mL^−1^) are prepared in order to study the effect of bacterial concentration on streamer formation. The approximate volume fractions of these cell concentrations are 10^−5^ and 2*10^−4^, respectively. The volume fractions are calculated by taking approximate single bacterial cell size of 1 μm by 2 μm (rod shape). These bacterial suspensions with different cell concentrations are denoted as *E. coli LC* and *E. coli HC*, respectively, in the rest of this report (as presented in Table 1): LC and HC denote low and high concentrations, respectively. SYTO 9 green-fluorescent nucleic acid stain (from Invitrogen) is used to observe the individual bacterial cells and the streamer formation phenomena during the filtration experiments.

The particle suspension used in this work consisted of monodisperse (2.3 μm in diameter) latex polystyrene microspheres (Invitrogen) dispersed in ultrapure water. The latex suspension used in the filtration experiments is obtained by diluting the stock suspension until a volume fraction of 10^−5^ (i.e., 1.6*10^6^ particles per ml) is reached. NaCl salt is used to change the ionic strength of the suspensions (154 mM) in order to make the suspension environment identical with the bacterial suspension for comparison purpose. The salt concentration 154 mM is inferior to the critical concentration for coagulation (CCC) which is observed by sedimentation tests to be above 200 mM. Prior to experiments, the latex suspensions are exposed to ultrasonic waves for 3 minutes in order to break down aggregates of particles, if any.

Pure suspensions of *E. coli* and polystyrene latex particles at identical salt concentration (i.e., 154 mM) are mixed in order to investigate the effect of *E. coli* presence with particles on the clogging dynamics. The volume fraction of ‘particles’ (both polystyrene and *E. coli*) is 10^−5^. The two pure suspensions are mixed just before the filtration experiments are performed in order to avoid the effect on the physiological conditions of the bacteria by the particle suspension. The SYTO 9 green-fluorescent nucleic acid stain is added to the bacterial suspension before the mixing. The mixture suspension is agitated by a vortex mixer for 30 seconds.

Filtration experiments are performed in pseudo-cross flow mode in the permeate side at constant flow rate of 20 μL/min (flow velocity of 0.170 m/s) by using Microfluidic Flow Control System (MFCS) and Flowell (from Fluigent). This flow velocity is well above the bacterial motility (~100 μm/s). Thus, the filtration experiments are mainly dominated by the hydrodynamic conditions and the role played by the bacterial motility is negligible. The filtration conditions are in laminar flow regime and the Reynolds number is 1.36. The pressure, flow rate and permeate volume are measured with time which are coupled with images from microscopic observations of the filtration experiments enabling both quantitative and qualitative analyses of the results. Fluorescent microscope (Zeiss) is used for the direct observations at magnifications ranging from 10X to 50X.

## Additional Information

**How to cite this article**: Sendekie, Z. B. *et al*. Bacteria Delay the Jamming of Particles at Microchannel Bottlenecks. *Sci. Rep.* **6**, 31471; doi: 10.1038/srep31471 (2016).

## Supplementary Material

Supplementary Information

Supplementary Movie

## Figures and Tables

**Table 1:** Main characteristics of the particles, bacteria and mixed suspensions (CFU is the colony-forming unit counting the number of viable bacteria).

Suspension type	Volume fraction	CFU/mL
Particles	10^−5^	n/a
*E. coli* LC	10^-5^	5*10^6^
*E. coli* HC	2*10^−4^	10^8^
Mix	*E. coli *= 10^−5^	5*10^6^
	Particles = 10^−5^	n/a
