# Supplementary material for: Bacteria Delay the Jamming of Particles at Microchannel Bottlenecks
Source: Sci Rep. 2016 Aug 11;6:31471. doi: 10.1038/srep31471 (PMC4980593; doi:10.1038/srep31471)
Supplement: Supplementary Information [file srep31471-s1.pdf]

Supplementary Information

Bacteria Delay the Jamming of Particles at Microchannel Bottlenecks

Zenamarkos Bantie SENDEKIE[a], Arthur GAVEAU[a], Rob LAMMERTINK[b], Patrice BACCHIN[a,*]

[a]*Laboratoire de Génie Chimique, Université de Toulouse, CNRS, INPT, UPS, Toulouse, France*

[b]*Soft Matter, Fluidics and Interfaces, MESA+ Institute for Nanotechnology, Faculty of Science and Technology, University of Twente, Enschede, The Netherlands.*

* corresponding author: patrice.bacchin@univ-tlse3.fr

1. Microfluidic Device

Microfluidic device fabricated by dry reactive-ion etching (DRIE) process has been used for this work. The microchannels are constituted of an array of 20 parallel channels having width of 5 µm. The microfluidic device assembly inside the chip holder and design of the chip (straight channels) are shown in Figure S1. The main channel and the microchannels have depth of about 20 µm. This microfluidic device is fabricated in MESA+ nano lab at University of Twente, The Netherlands.

The chip is designed for cross flow separation or fractionation of 'particles' (biological and colloidal particles). The microchannels for the first separation have widths of 10 µm and the second separation section is 5 µm. We use this design in a pseudo-cross-flow mode in the permeate side (figure S1c inset image) for this experimental work: the inlet leading to the first microchannels with widths of 10 µm and the retentate outlet from this separation section are

closed and the particle and bacterial suspensions flow through the 5 µm wide microchannels with one inlet and one outlet.

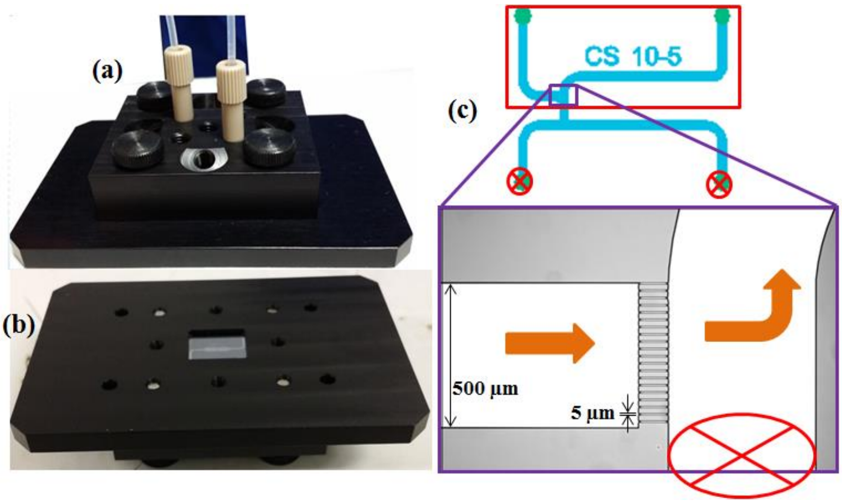

Figure S1. Microfluidic device used for the filtration experiments: a) top side of the chip holder; b) bottom side of the chip holder in which the chip is assembled; c) chip design used in this work and the inset image is microscopic image of the filtering section. C denotes cross-flow, S straight channels, 5 is the channel width in µm. The width of the main feeding channel is 500 µm.

2. Analysis of the average thickness of particle/bacteria deposit

The image analysis (ImageJ software) enabled us for the quantification of the surface occupied by the deposited particles and bacteria present in the microsystem. The average deposit thickness, *e*, can be estimated through the analysis of the mean grey value:

$$\frac{e}{e_{box}} = \frac{Gv - Gv_{init}}{Gv_{deposit} - Gv_{init}}$$

Where e_{box} is the dimension (in the deposit thickness direction) of the zone of interest, Gv is the mean grey value for a given time, Gv_{init} is the initial mean grey value and $Gv_{deposit}$ is the mean grey value of the deposit.

For the mixture of bacteria and particle, the kinetics for the formation of particle deposit (through the particle signal) and of the bacteria (through the bacteria fluorescence signal) are plotted in the figure S2.

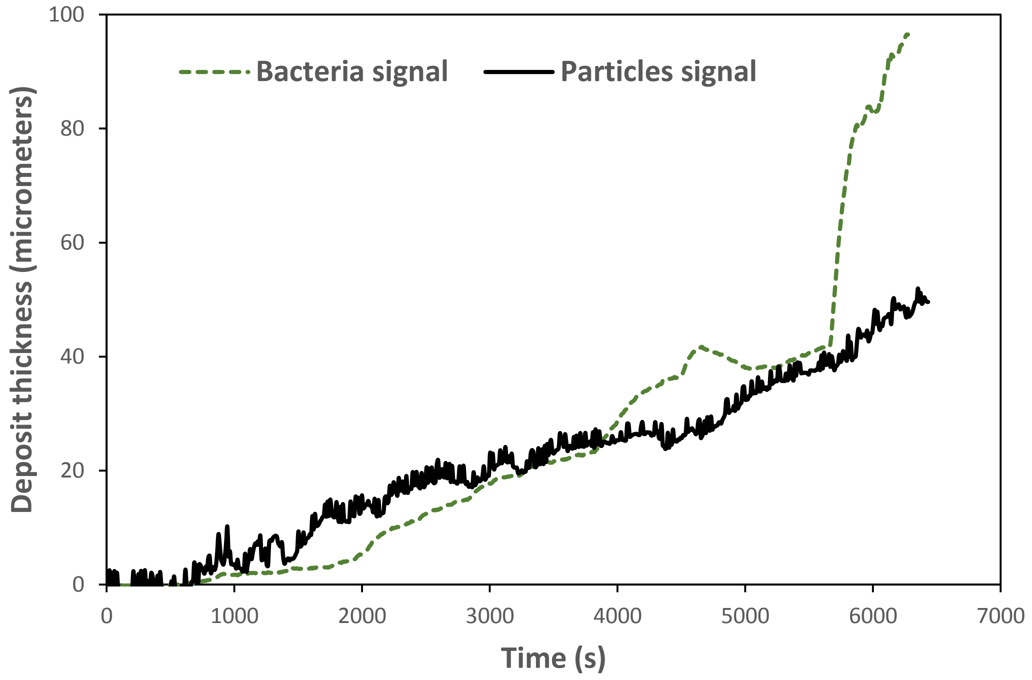

Figure S2. Variation of the average deposit thickness with time deduced from the particles signal (black and white channel) and from the bacterial signal (green channel)

The appearance of bacteria and particles is rather well correlated. However, the analysis shows that the accumulation of particles seems to start before the increase of the fluorescent signal (between 1000 s and 3000 s). Furthermore, at the end of the experiment, it can be noted that the fluorescent signal is increasing a lot around 5700 s. This increase is correlated to the increase of pressure presented in the figure 4 of the paper that corresponds to the complete blocking of all the microchannels by the particle/bacteria mixture. By analysing

the images, it can be seen that the total flow blocking induces the greening of the deposit thickness probably due to the percolation of bacteria in the dense deposit. At the end, it can be seen that the green signal (due to the bacteria presence) is very well correlated to the pressure increase due to the clogging (figure S3). This correlation could be due to the fact that the capture of the bacteria in the deposit is directly linked to the percolation of the water through the deposit.

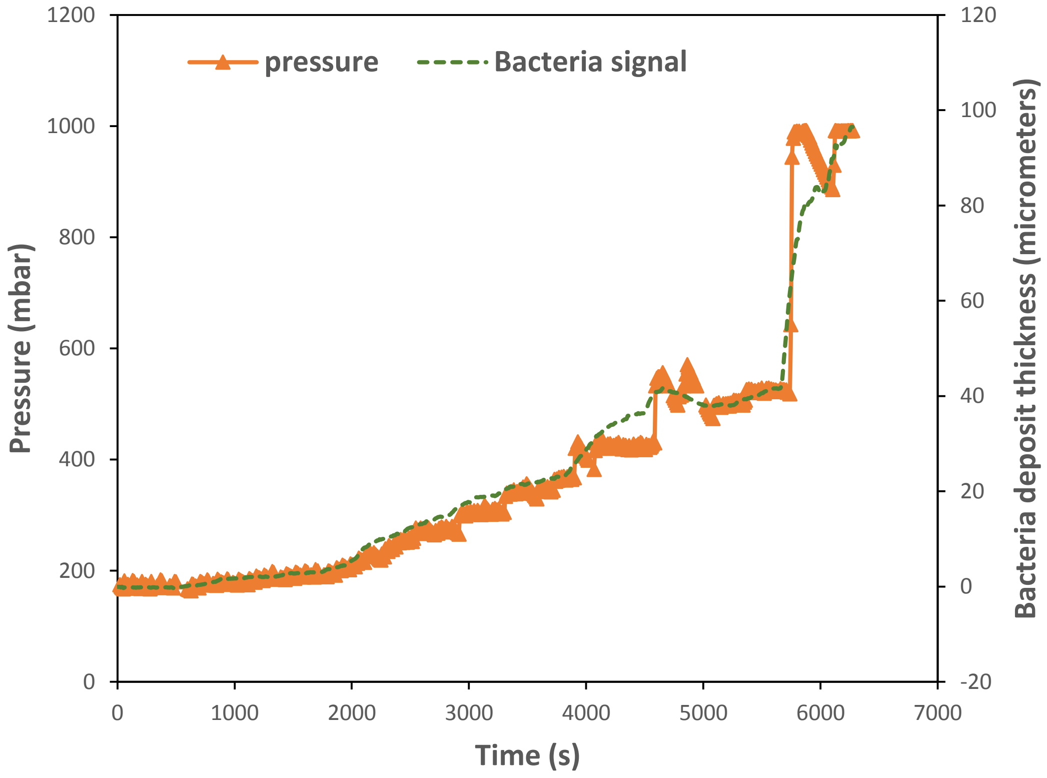

Figure S3. Correlation between the pressure increase and the average bacterial deposit thickness during filtration of particle/bacteria mixing

3. Video

Video : Video showing the filtration of bacteria (in green)/particles (in red) mixture (shown in figure 3 as an image montage).
